# Cellular Immunotherapies for Multiple Sclerosis: Mechanistic Insights and Clinical Advances

**DOI:** 10.3390/ijms27020585

**Published:** 2026-01-06

**Authors:** Vasily Kurilin, Marina Fisher, Irina Obleukhova, Sergey Sennikov

**Affiliations:** Laboratory of Molecular Immunology, Federal State Budgetary Scientific Institution “Research Institute of Fundamental and Clinical Immunology” (RIFCI), Novosibirsk 630099, Russia; vkurilin@niikim.ru (V.K.); msolshanova@gmail.com (M.F.); obleukhova.irina@yandex.ru (I.O.)

**Keywords:** cell therapy, multiple sclerosis, HSC, T cell, Treg, CAR, TCR

## Abstract

Multiple sclerosis (MS) is a chronic, heterogeneous, multifactorial, immune-mediated neurodegenerative disease of the central nervous system that affects the working-age population. Its development is influenced by both genetic and environmental factors. A pathological hallmark of MS is the formation of demyelinating lesions in the brain and spinal cord, which are associated with neuronal damage caused by autoaggressive immune factors (T cells, B cells, and myeloid cells). Focal lesions are believed to be caused by the infiltration of immune cells into the central nervous system (CNS) parenchyma with concomitant tissue damage. Multiple sclerosis represents a significant social problem due to the high cost of available treatments, as well as the deterioration of employment prospects and job retention for both patients and their caregivers. Advances in MS diagnostic methods have enabled disease detection at early stages and correction of immune response impairments. Concurrently, treatments for MS patients are actively being studied, with the ongoing development of novel methods for targeted and cellular immunotherapy. This review primarily discusses approaches to cellular immunotherapy and methods of influencing the cellular arm of immunopathogenesis in multiple sclerosis.

## 1. Introduction

Modern treatment of multiple sclerosis combines pharmacological, physiotherapeutic, and rehabilitative approaches aimed at reducing disease activity, alleviating symptoms, and improving quality of life [1]. Disease-modifying therapies (DMTs), which modulate the immune response and attenuate neuroinflammation, thereby slowing disease progression, remain the cornerstone of management [2]. However, many patients exhibit an inadequate or variable response, and current DMTs have a limited impact on neurodegeneration and progressive forms of the disease [3,4,5,6].

Long-term use can also lead to the development of resistance and adverse events, including immunosuppression, osteoporosis, and gastrointestinal disorders [7,8].

Given the advances in cell therapy for oncological diseases, it is pertinent to apply these technologies to the treatment of autoimmune disorders, particularly multiple sclerosis. The development and application of cellular immunotherapy for MS is driven by the need to restore immune tolerance to self-antigens of the CNS (such as myelin basic protein (MBP), proteolipid protein (PLP), and myelin oligodendrocyte glycoprotein (MOG)). This therapy aims for targeted modulation of the immune response: suppressing autoreactive T cells (including Th1 and Th17 subtypes that produce proinflammatory cytokines such as IFN-γ and IL-17), inducing anergy (functional inactivation) or apoptosis of these cells, and activating regulatory mechanisms, such as Treg cells (CD4+ CD25+ Foxp3+), Tr1 cells (producing IL-10), and Breg cells. Consequently, an anti-inflammatory environment is created, characterized by increased production of IL-10, TGF-β, and IL-35, which contributes to suppressing the autoimmune attack, reducing inflammation in the CNS, and potentially promoting tissue repair. The advantages of cellular immunotherapy include high specificity (minimizing systemic immunosuppression), reduced risk of side effects, and the potential for long-term tolerance, which is particularly important for progressive forms of MS where conventional drugs are less effective. The aim of this review is to systematize and analyze the existing knowledge on cell therapy for multiple sclerosis to understand future prospects for the development of this technology.

## 2. Cellular Therapy Approaches for Multiple Sclerosis

**Figure 1:**
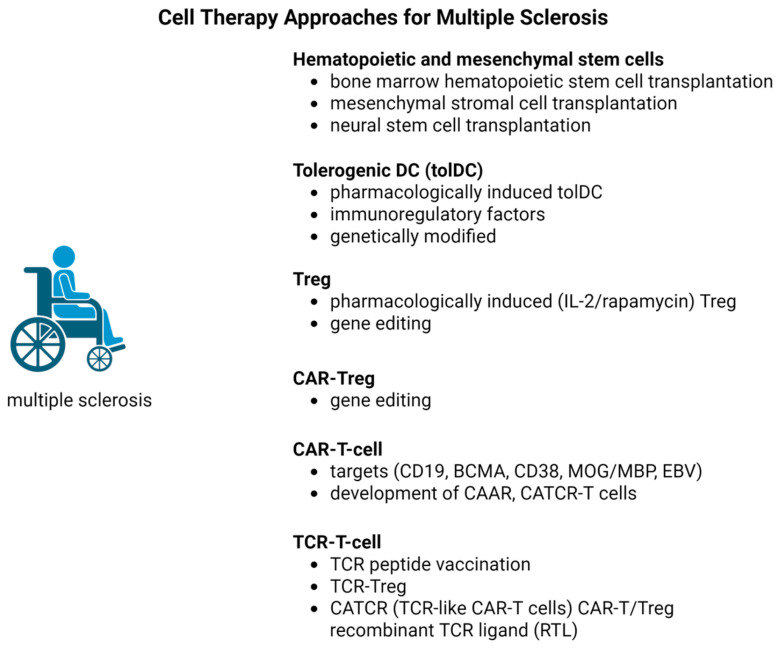
Modern approaches to cellular immunotherapy for multiple sclerosis. Illustration classifying current and emerging cellular immunotherapies for MS. Therapies are grouped by mechanism: immune reset (e.g., AHSCT), immunomodulation and neuroprotection (e.g., MSCs and tolDCs), tolerance induction (e.g., adoptive Treg transfer), and antigen-specific engineering (e.g., CAR-Tregs, CAR-T cells targeting B cell lineages, and TCR-T cells).

Despite their proven efficacy in suppressing inflammatory processes and preventing clinical relapses, existing disease-modifying therapies have demonstrated limited therapeutic impact on progressive forms of multiple sclerosis. A critical unresolved issue remains their inability to significantly slow or halt the neurodegenerative processes underlying disease progression. In this context, the development of targeted therapeutic strategies aimed at key elements of MS pathogenesis, including pathogenic effector cells (T and B lymphocytes), regulatory T cells (Tregs), and specific autoantigens, is of particular relevance. Modern experimental in vitro and in vivo studies have focused on evaluating the therapeutic potential of major directions in cellular immunotherapy for MS: hematopoietic stem cells, tolerogenic dendritic cells, regulatory T cells (Tregs), CAR-T cells, and TCR-T cells [9,10,11] (Figure 1).

Each of these approaches possesses a unique mechanism of action and the potential to modulate immune processes in MS, opening new prospects for developing effective treatments, especially for progressive forms of the disease (Table 1).

### 2.1. Hematopoietic and Mesenchymal Stem Cells

The refinement of therapies that target specific immune cells or generate targeted cell populations is driven by progress in understanding the immunopathogenesis of multiple sclerosis and advancements in biotechnology. One of the most common approaches in cellular therapy is the use of hematopoietic stem cells. This method is considered a direction for treating neurological diseases because of the ability of stem cells to regenerate damaged nervous system tissues [12].

Autologous hematopoietic stem cell transplantation (AHSCT) is used in MS therapy, particularly in patients resistant to standard immunotherapy [12]. Initially, developed for treating hematological malignancies with poor prognoses, AHSCT has been studied over the past two decades in the context of severe autoimmune pathologies refractory to conventional treatments [13]. The theoretical rationale for this approach is based on the concept of “resetting” the immune system by eliminating autoreactive T and B cells through immunoablative conditioning therapy (e.g., chemotherapy with antibodies), followed by reinfusion of autologous CD34+ hematopoietic stem cells. This process leads to the reconstruction of the T-cell repertoire, an increase in regulatory T cells (Tregs), and a reduction in proinflammatory Th17 cells, thereby restoring immune tolerance. Consequently, inflammatory activity is suppressed, biomarkers (such as neurofilament light chain, NfL) normalize, and brain atrophy slows. This leads to the modification of T-cell responses and other immune reactions, potentially improving the clinical course of autoimmune disease, including pathological immune responses in MS [14] (Table 1).

Following initial clinical observations, such as the study by Fassas et al. [15], MS has become one of the most common autoimmune diseases for which AHSCT is applied [16]. The traditional sources of HSCs are bone marrow or peripheral blood. The latter is preferred because it is a less invasive collection method; however, the low concentration of circulating stem cells requires preliminary mobilization from the bone marrow using cyclophosphamide or growth factors such as granulocyte colony-stimulating factor (G-CSF). The combined use of cyclophosphamide and G-CSF is considered optimal, as cyclophosphamide reduces the risk of MS exacerbation induced by G-CSF and contributes to reducing T-cell numbers during apheresis [17].

After collection, HSCs undergo processing, including positive selection of CD34+ cells for lymphocyte depletion and/or purification via anti-lymphocyte antibodies (e.g., anti-CD52) or cytotoxic agents [18]. The key markers for identifying and isolating HSCs are the surface antigens CD34 and Thy-1, which are characteristic of early progenitor cells [18]. The final stage involves conditioning the recipient’s immune system, aimed at its suppression or elimination, ensuring successful repopulation by the transplanted HSCs and preventing the recovery of the original autoreactive immune system [19].

Clinical studies have demonstrated the ability of AHSCT to normalize the expression of immunoregulatory genes, leading to the restoration of immune system balance [20,21]. This approach induces significant changes in the immune regulatory compartment, including a transient increase in the population of regulatory FoxP3+ T cells [19]. In multiple sclerosis, AHSCT promotes remodeling of the CD4+ cell pool, reduces encephalitogenic potential by decreasing the number of peripheral Th17 and Tc17 lymphocytes, suppresses antigen-presenting function, and increases the number of immunoregulatory cells [19].

However, an absolutely positive effect is not always observed. Histopathological analysis of samples from five MS patients who underwent AHSCT revealed signs of ongoing demyelination. Inflammatory infiltrates, predominantly composed of cytotoxic CD8+ T cells associated with axonal degeneration, have been observed in lesions [22]. These data indicate the persistence of neurodegenerative processes despite immunosuppressive therapy, which is correlated with clinical observations of continued disease progression and magnetic resonance imaging (MRI) activity in AHSCT studies [22]. Additionally, rapid brain volume loss was recorded in the first months post-transplantation, followed by a decrease in atrophy rates after 2 years. The initial volume loss may be related either to the regression of postinflammatory edema or to the toxic effects of the conditioning regimen [23]. The described negative dynamics of MS treatment may be due to the individual characteristics of the course of the disease in the examined patients, or the presence of concomitant diseases that could indirectly affect the effectiveness of therapy [23].

Despite this, AHSCT demonstrates high efficacy in reducing clinical relapse rates, comparable to or exceeding those of the most potent modern drugs (alemtuzumab, natalizumab). However, owing to the potential risks of the procedure, including increased mortality, determining the place of AHSCT in the treatment algorithm for relapsing–remitting MS requires further investigation [20,21].

In addition to hematopoietic stem cells, bone marrow contains a heterogeneous population of mesenchymal stromal cells (MSCs) [24]. These cells possess immunosuppressive properties and are capable of modulating immunity, promoting remyelination (by differentiating into oligodendrocytes or stimulating endogenous precursors), and providing neuroprotection through anti-apoptotic effects and stimulation of angiogenesis [25,26,27].

**Figure 2:**
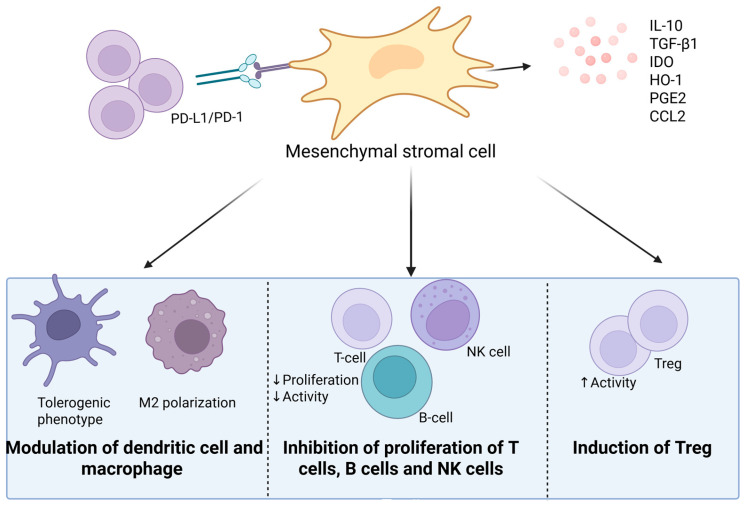
Immunosuppressive properties of MSCs. MSCs secrete prostaglandin E2, TGF-β, IDO, IL-10, NO, CCL2 and express PD-L1 and PD-L2. These factors lead to inhibition of proliferation of T-, B-, and NK-cells, polarization of macrophages toward the M2 phenotype, reduced maturation and activation of dendritic cells, and induction of regulatory T-cells.

The most significant property of MSCs for the therapy of autoimmune diseases is their immunosuppressive effect (Figure 2). This effect is mediated through several mechanisms. MSCs secrete soluble factors (PGE2, TGF-β, IDO, IL-10) that suppress T-cell proliferation and activation, induce their apoptosis, promote the differentiation of regulatory T-cells, inhibit T-cell proliferation, and induce a Th2-mediated response [28]. Furthermore, they modulate the functions of NK cells, B cells, and dendritic cells (by suppressing their maturation and reducing the expression of CD83+, CD86+, and HLA-DR [29]), and polarize macrophages toward an anti-inflammatory phenotype. Additionally, MSCs increase the proportion of CD4+CD25+ Treg cells [30], express surface receptors such as PD-L1, which inhibits T-cell activation via interaction with PD-1, produce nitric oxide (NO), and secrete CCL2, which inhibits immune cell migration and promotes macrophage polarization toward an M2 phenotype. Overall, the immunosuppression mediated by MSCs is achieved through a combination of these mechanisms and may vary depending on the microenvironmental conditions.

Furthermore, MSCs also possess a range of neuroprotective properties. They promote oligodendrogliogenesis and protect neurons from oxidative stress, partly through the secretion of various neurotrophins (brain-derived neurotrophic factor and nerve growth factor). Consequently, MSCs are potential therapeutic agents for MS, limiting inflammation; protecting axons, neurons, and glia; and promoting remyelination [31].

Systemic transplantation of autologous or allogeneic MSCs in relapsing–remitting or progressive models of experimental autoimmune encephalomyelitis (EAE) leads to reduced activation of T and B cells, accompanied by clinical and histological improvements, decreased numbers of inflammatory lesions, and reduced axonal loss with preserved myelin structure [32].

The largest studies on therapeutic MSC transplantation have been conducted in patients with hematological malignancies, breast cancer, ischemic heart disease, and graft-versus-host disease (GVHD) [33]. The results revealed relative safety and a low level of side effects and adverse events from cell therapy. The demonstrated safety and therapeutic effects of MSCs, combined with experimental data from laboratory animal and/or cell models, provide a rationale for clinical trials in MS. In 2008–2009, Connick et al. studied ten MS patients and conditionally healthy donors and successfully isolated and characterized their MSCs in vitro [34]. MSC infusion in MS patients improved visual function, stabilized disease progression, and/or improved the Expanded Disability Status Scale (EDSS) score [35]. Currently, the international multicenter randomized double-blind placebo-controlled crossover phase II study MESEMS (Mesenchymal Stem Cells for Multiple Sclerosis), involving 15 scientific and clinical centers in nine countries, is ongoing. Since the start of the MESEMS program (2012), 69 patients infused with MSCs derived from bone marrow have been studied. Transplantation of MSCs was shown to be safe regardless of the route of administration; however, clinical and instrumental parameters did not demonstrate therapeutic efficacy, which may be related to patient heterogeneity and the use of immediately thawed MSCs without prior short-term cultivation [36].

Simultaneously, experimental research is underway to improve the MSC-based approach. This includes priming cells in various ways in culture before infusion or genetic modification of MSCs to presumably improve aspects of their function, including survival, neuroprotective or restorative function, or homing to specific target tissues [37].

Neural stem cells, or neural precursor cells (NPCs), also possess neuroprotective properties. They can differentiate into oligodendrocytes or astrocytes to directly facilitate remyelination and neurogenesis. These cells migrate to lesion sites, where they secrete LIF and BDNF to reduce T-cell infiltration and modulate macrophage metabolism, thereby preventing axonal degeneration. This is supported by studies using experimental autoimmune encephalomyelitis (EAE) models, where transplantation of NPCs led to a significant reduction in clinical disease severity and a decrease in pathological inflammatory markers [38]. In a viral model of demyelinating disease, intraspinal transplantation of human embryonic stem cell-derived NSCs led to sustained clinical recovery [39].

There is also work on obtaining MSCs from tissues other than bone marrow. “PDA-001” is a preparation of mesenchymal-like cells derived from healthy human placental tissue. It caused dose-dependent prevention of EAE induction and, in induced EAE, reduced disease progression and severity [40]. “PDA-001” has also been investigated in a multicenter randomized double-blind study in patients with relapsing–remitting and secondary progressive MS—the first therapeutic study investigating the human placenta as a source of therapeutic stem cells. In this study, 81% of patients were also taking at least one licensed MS drug, making the determination of treatment effect complex; however, the administration of “PDA-001” in MS patients proved to be safe and feasible. “PDA-001” may have significant advantages as an alternative cell source; the term fetal placenta is a rich source of nonembryonic cells, and scalable production is also possible [40].

Clinical studies have confirmed the safety of autologous MSCs in MS. The MESEMS study (NCT01854957) demonstrated the stabilization of EDSS values, improved visual functions [41,42], and the absence of serious adverse events [43].

The risks associated with the use of mesenchymal stromal cells (MSCs) in the treatment of multiple sclerosis (MS) are generally considered to be low. Phase I/II clinical trials and systematic reviews up to 2025 confirm a favorable safety profile, with serious adverse events being rare and therapy well-tolerated by patients with various forms of MS (relapsing, secondary progressive). However, potential risks do exist and can be categorized as immediate, short-term, and theoretical long-term risks. The most common adverse events are mild and transient, typically related to the administration procedure (intravenous or intrathecal), such as headache, fever, urinary or respiratory tract infections, and local infusion-site reactions. These effects are usually self-limiting and do not necessitate therapy discontinuation. Large-scale reviews (e.g., the MESEMS study and meta-analyses) have not recorded serious complications like cytokine release syndrome or anaphylaxis [36].

The main challenge lies in variable or modest clinical efficacy. In some trials, including placebo-controlled studies, MSCs did not significantly reduce inflammatory activity or disability progression, particularly in active forms of MS. Overall, as of 2025, MSC therapy is considered one of the safest cell-based approaches for MS, with its primary limitation being its unproven efficacy for consistent therapeutic benefit [36].

Future research focuses on optimizing dosing regimens, combination strategies, and patient stratification to minimize even minimal risks. Building on this work, future developments may advance toward the use of stem cell-derived secretomes, particularly MSC-derived exosomes, for cell-free therapeutic applications.

### 2.2. Tolerogenic Dendritic Cells

Dendritic cells (DCs), which perform dual functions, play a central role in the immunopathogenesis of multiple sclerosis (MS). Their proinflammatory activity is due to increased expression of costimulatory molecules (CD80/CD86) and proinflammatory cytokines (IL-12, IL-23, and TNF-α) [44,45], enhanced migration to the CNS via the chemokine receptors CCR5/CCR7 [46,47], induction of a Th17 response, and disruption of regulatory mechanisms [48].

Dendritic cells (DCs) play a central role in the immunopathogenesis of multiple sclerosis (MS), exhibiting both proinflammatory and regulatory functions. Their contribution to the development of autoimmune inflammation is attributed to the increased expression of costimulatory molecules CD80/CD86 and the secretion of proinflammatory cytokines, including IL-12, IL-23, and TNF-α [44,45], enhanced migration into the central nervous system (CNS) via chemokine receptors CCR5 and CCR7 [46,47], as well as the induction of Th17 responses and the impairment of immune regulatory mechanisms [48].

Concurrently, DCs possess a significant tolerogenic potential, implementing mechanisms of central and peripheral tolerance. The maintenance of a tolerogenic state is mediated through the PD-L1/PD-1 and CTLA-4 signaling axes [49,50], and by the induction of regulatory T-cells (Foxp3+ Tregs and Tr1) under the influence of TGF-β and IL-10 [51,52]. Consequently, tolerogenic dendritic cells (tolDCs) are considered a promising therapeutic approach, enabling the induction of antigen-specific tolerance without causing generalized immunosuppression. Studies have shown that autologous DCs loaded with myelin antigens (MOG, MBP) can stimulate Tregs and suppress autoreactive T-cells via IL-10, TGF-β, and inhibitory molecules such as PD-L1.

**Figure 3:**
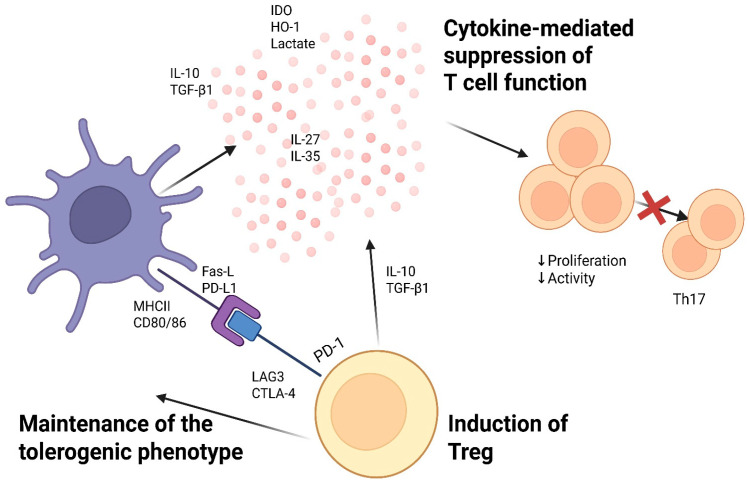
Mechanisms of the immunosuppressive and tolerogenic actions of dendritic cells. TolDCs maintain a tolerogenic phenotype through low expression of costimulatory molecules (CD80/CD86), antigen presentation in the context of MHC II, and expression of inhibitory ligands such as Fas-L and PD-L1. Through the production of anti-inflammatory cytokines (IL-10, TGF-β1, IL-27, IL-35), as well as metabolic factors (IDO, HO-1, lactate), tolDCs suppress the proliferation and functional activity of effector T cells, including Th17 cells. At the same time, tolDCs induce the differentiation of regulatory T cells (Tregs), which, by expressing PD-1, CTLA-4, and LAG-3, further inhibit antigen-presenting cells and promote the secretion of IL-10 and TGF-β, thereby establishing a positive feedback loop that sustains immune tolerance.

During early immune development, DCs are involved in establishing central tolerance by presenting tissue-specific autoantigens expressed by medullary thymic epithelial cells, thereby facilitating the negative selection of high-affinity autoreactive thymocytes. Subsequently, tolDCs maintain peripheral tolerance by inducing anergy, apoptosis, or functional hyporesponsiveness in autoreactive T-cells (Figure 3) that have escaped central control. These effects are achieved through antigen presentation in the context of MHC II in the absence of adequate co-stimulation and without the activation of proinflammatory cytokines.

Tolerogenic dendritic cells (tolDCs) are characterized by resistance to proinflammatory stimuli, low expression of maturation marker CD83 and costimulatory molecules CD80/CD86, as well as increased secretion of IL-10 and TGF-β [53,54]. The interaction of PD-L1 on DCs with PD-1 on T-cells leads to the transmission of inhibitory signals, suppression of T-cell receptor signaling pathways, and the development of clonal anergy [55]. Fas-L expression on tolDCs promotes the induction of T-cell apoptosis, while elevated levels of inhibitory receptors on DCs enhance the activation of regulatory T-cell populations [56].

In addition to contact-dependent mechanisms, tolDCs exert immunosuppressive effects via contact-independent pathways associated with the production of anti-inflammatory cytokines (IL-10, TGF-β, IL-27, IL-35) and metabolic factors, including indoleamine 2,3-dioxygenase (IDO), heme oxygenase-1 (HO-1), and lactate. IDO catalyzes the breakdown of tryptophan to generate kynurenines, which suppresses T-cell proliferation and promotes their differentiation into regulatory cells. HO-1, in turn, exerts an anti-inflammatory effect and contributes to the maintenance of the Treg pool, while increased lactate production reduces glycolysis, activation, and proliferation of effector T-cells [57].

Of particular importance is the ability of tolDCs to induce various subtypes of regulatory T-cells, including CD4+Foxp3+ Tregs and CD4+IL-10-producing Tr1 cells [58]. This process is mediated both by the production of anti-inflammatory cytokines that stimulate the expression of the transcription factor Foxp3 in naive T-cells and their subsequent differentiation [59], and as a result of repeated stimulation of T-cells by dendritic cells in the absence of maturation signals [60]. Induced Tregs suppress effector T-cells and natural killers through the secretion of IL-10 and TGF-β [61,62], compete for IL-2 via high expression of CD25 [63], and inhibit the function of antigen-presenting cells through CTLA-4, PD-1, and LAG3 by blocking CD80/CD86 and binding to MHC II on DCs [64].

Currently, tolerogenic DCs are generated in vitro; in most protocols, DCs are differentiated from peripheral blood monocytes via pharmacological agents (dexamethasone, vitamin D3 (1,25(OH)2D3), and rapamycin), immunoregulatory factors (anti-inflammatory cytokines (TGF-β and IL-10), NF-κB inhibitors, and apoptotic cells), genetic approaches involving the use of antisense oligonucleotides to suppress costimulatory molecules, and transduction via lentiviral vectors encoding IL-10 and disease-associated peptides [53,54,56,57].

Although each of these methods involves different molecular mechanisms, the common phenotypic features of the resulting tolDCs include a more immature phenotype characterized by low expression of the maturation marker CD83; low expression of the costimulatory receptors CD80 and CD86; reduced secretion of proinflammatory cytokines such as IFN-γ, IL-1, IL-6, IL-12, and TNF-α; increased production of anti-inflammatory cytokines such as IL-10 and TGF-β; and the ability to induce Treg differentiation [65,66]. The result is the presentation of autoantigens to T cells without concomitant costimulation, leading to reduced activation of effector T cells and immune tolerance. Importantly, tolDCs must also retain their tolerogenic phenotype upon subsequent exposure to proinflammatory stimuli [67].

Peripheral blood monocytes are the primary source for generating dendritic cells in vitro. In this context, monocytes from MS patients have been characterized, revealing a shift toward proinflammatory properties characterized by changes in the AhR and NF-κB pathways. This affects the efficiency of generating tolDCs, which are characterized by lower tolerogenic properties than those of conditionally healthy donors. In vitro and in vivo studies have shown that, unlike monotherapy, tolDCs obtained from animals with induced experimental autoimmune encephalomyelitis (EAE) have a significant effect only in combination with a drug (dimethyl fumarate) [68]. In multiple sclerosis, dysregulation of both the frequency and function of DCs is observed. Conventional DCs adopt a more immunogenic phenotype with increased production of the proinflammatory cytokines IFN-γ, TNF-α, IL-6, IL-12, and IL-23 [69] and expression of the chemokine receptors CCR5 and CCR7, which are associated with increased migration to CCR5 and CCR7 ligands [70] (Table 1).

To improve the efficacy of tolerogenic DC application, delivery methods are being optimized—intradermal/intralymphatic administration to improve migration to lymph nodes, the use of CCR5-expressing tolDCs to cross the blood–brain barrier [71], and the search for new target autoantigens beyond myelin peptides (MBP, MOG, PLP). Tolerogenic DNA vaccines involve the delivery of DNA encoding autoantigens associated with autoimmune disease. This can be induced by incorporating plasmids with reduced CpG content to suppress innate immune activation. A phase I/II trial in MS patients using a DNA vector encoding myelin basic protein (MBP) led to reduced antigen-specific activity of IFN-γ-producing CD4+ T cells [72]. mRNA vaccine technology has also been used in a recent EAE treatment study. The administration of nanoparticles modified with 1-methylpseudouridine and carrying mRNA encoding the disease-specific peptide MOG35-55 led to antigen presentation on splenic CD11c+ antigen-presenting cells without costimulatory signals, suppressing EAE and T effector cells while stimulating Tregs [73].

Notably, there is a potential risk with DNA/mRNA-based immunotherapy approaches, particularly peptides, which involve exacerbation of the disease course if peptides are presented against a background of inflammation. For example, preclinical studies using nanoparticles conjugated with the MOG35-55 peptide targeted to cells expressing MHC class II (MHC-II), which were administered to mice with active EAE, triggered a “cytokine storm” reaction [74]. In a phase II clinical trial of an altered peptide ligand of myelin basic protein (MBP83-99), instead of inducing regulatory myelin-specific T cells, the peptide had an encephalitogenic effect and led to the exacerbation of MS in a subgroup of patients [75].

Currently, several open-label dose-escalation phase I studies of tolDC-based therapy are being conducted in patients with active relapsing–remitting MS (RRMS) and primary progressive MS (PPMS) up to EDSS 6.5, without background disease-modifying therapy. For cellular therapy, autologous DCs are cultured with vitamin D3 to induce a tolerogenic phenotype and then primed with seven myelin peptides (MBP13-32, MBP83-99, MBP111-129, MBP154-170, MOG1-20, MOG35-55, and PLP139-154). Six doses were administered intradermally in the neck or directly into the cervical lymph nodes. The primary outcomes were safety and tolerability; cellular responses, including lymphocyte populations, myelin-specific T-cell reactivity, and cytokine production, as well as clinical and radiological outcomes, were assessed (NCT02618902, NCT02903537).

Clinical studies of MS therapy based on human tolerogenic dendritic cells loaded with neural tissue peptides have been conducted. Compared with untreated mature DCs, trials of therapy with autologous tolDCs treated with dexamethasone and loaded with seven peptides derived from myelin (MBP13-32, MBP83-99, MBP11-129, MBP146-170, MOG1-20, MOG35-55, and PLP139-154) in MS patients (progressive course) revealed increased expression of the MERTK protein, a marker of tolDCs. Treatment was safe and well tolerated with increased peptide-stimulated IL-10 production, but there were no differences in T-cell proliferation measured by ex vivo stimulation with individual peptides. A trend towards increased Tr1 cell frequency and a significant decrease in CD8 memory cells was observed. The participants had predominantly progressive disease (7 out of 8 MS patients), the HLA haplotypes were heterogeneous, and one participant was HLA-DRB1*1501-positive (NCT02283671).

A placebo-controlled phase II study of tolDC therapy with immunogenic peptides versus placebo in RRMS has been conducted (NCT04530318). The use of a “cocktail” of myelin peptides has not definitively shown that myelin-derived proteins are pathogenic or specific to MS; thus, investigating additional autoantigens is necessary.

Further patient stratification may also be required on the basis of the variability of disease-specific autoantigen profiles between individuals, potentially linked to the HLA genotype, which influences antigen presentation. There is a direction for more precise stratification on the basis of other MS risk genes, such as the MERTK allotype, in combination with the HLA haplotype. The MERTK rs7422195 SNP was found to either increase or decrease the risk of developing MS, depending on the HLA-DR15 status. Homozygosity for the minor A allele at rs7422195 was associated with higher MERTK expression in a larger fraction of monocytes, which are precursors of DCs, in a group of healthy individuals among whom MS development was not excluded [76].

However, this genotype-based stratification was lost in patients with established disease, where compensatory responses may lead to an increase in MERTK-expressing monocytes in patients homozygous for the major G allele at rs7422195. Furthermore, DR15-positive individuals with MS had significantly lower proportions of MERTK-expressing monocytes than DR15-negative individuals did, which further decreased during relapse [76]. The subsequent differentiation of MERTK-expressing CD14+ monocytes is important for maintaining immune homeostasis, with human MERTK-expressing DCs suppressing T-cell proliferation and MERTK-mediated efferocytosis of myelin debris by macrophages [77]. Thus, the role of MERTK in mediating immune tolerance and its association with HLA-DR15 in MS risk suggest that the MERTK genotype and its expression profile on innate immune cells may influence the efficacy of tolDC therapy in MS.

Ideally, the route of administration should ensure that the maximum number of tolDCs reaching the lymph nodes where T cells reside. The methods used for intravenous, intradermal, and subcutaneous administration have been described. DC migration to the lymph nodes was more effective after intradermal than after subcutaneous injection [78]. In MS, neural tissue and myelin antigens can drain to the cervical lymph nodes [79], which may be the initial site of antigen presentation and thus a suitable site for tolDC administration [80].

The administration of tolDCs near the affected tissue is another option, e.g., intra-articular injection in patients with inflammatory arthritis, but in MS, this option must be balanced with patient acceptability. Intrathecal (intraspinal) administration is a serious consideration for MS patients and is therefore less preferable than intradermal administration [78].

The blood–brain barrier is a natural obstacle to the migration of administered cells. Chemokines can counteract this effect by promoting DC migration to sites of inflammation. Elevated levels of CCR5 ligands are observed in MS lesions, and circulating DCs in MS patients have been shown to have increased CCR5 expression, suggesting that CCR5 may facilitate DC infiltration into inflamed CNS tissues [81].

However, preclinical studies have demonstrated that the positive action of tolDCs may not be associated with their passage through the blood–brain barrier. Cell tracking methods were used to monitor labeled MOG40-55 peptide-loaded tolDCs after intravenous administration in experimental autoimmune encephalomyelitis. The administered tolDCs primarily accumulated in the lungs, liver, and spleen. Although only a weak transient signal was recorded in the brain, this did not prevent improvement in the disease course [82].

Blood–brain barrier permeability also increases during periods of inflammatory relapse and may increase the transport of tolDCs into the CNS. Therefore, the timing of treatment relative to periods of inflammatory activity must be considered. The administration of MS autoantigens into a proinflammatory environment may lead to disease exacerbation. This risk is mitigated by the presentation of MS autoantigens by a tolerogenic cell. In contrast, in progressive disease, inflammation becomes compartmentalized within the CNS, which may reduce the transport of peripherally administered tolDCs into the CNS [83].

Prophylactic administration of tolDCs before clinical symptoms appear, while shown in animal studies to prevent or delay EAE, is usually not feasible in MS. The beneficial effect of MOG40-55 peptide-loaded tolDCs in EAE lasts only five to six days, requiring repeated administration to maintain efficacy [78].

However, long-term use (over 8 months) of previously cryopreserved tolDCs allowed for a reduction in EAE severity, avoiding the need for frequent leukapheresis procedures [84]. A phase I clinical trial in MS/neuromyelitis optica spectrum disorder (NMOSD) patients revealed that three administrations of tolDCs at 2-week intervals led to a significant increase in peptide-stimulated IL-10 production by week 12 [78].

Cryopreservation of cells also shows promise: analysis of the viability of human mononuclear cells (MNCs) after 12 years of storage revealed that at least 50% of cells were recovered, with their viability being ≥90% [84]. Nevertheless, additional studies are needed to assess the possibility of generating tolDCs from long-term cryopreserved peripheral blood MNCs [84].

Progress in this direction has been achieved with the development of minimal reporting guidelines for the manufacturing process of tolerogenic APCs, known as the Minimal Information about Tolerogenic Antigen-Presenting Cells (MITAP), by the AFACTT consortium (Action to Focus and Accelerate Cell-based Tolerance-inducing Therapies) [85,86]. The most important components of tolDC production highlighted by the AFACTT are the cell status prior to manufacturing, DC differentiation and tolerogenicity induction processes, cell characteristics prior to administration or use in experimental assays, and general protocol characteristics, including regulatory approvals and GMP guidelines. With the growing number of clinical trials, the development of harmonized reporting criteria and quality standards becomes important to increase the likelihood that the cell product will be safe and effective.

The use of tolerogenic DCs is one of several approaches to induce immune tolerance and a promising approach for the personalized treatment of autoimmune diseases such as multiple sclerosis.

### 2.3. Tregs

Regulatory T cells (Tregs) have potent immunosuppressive effects and play key roles in regulating the immune system, maintaining self-tolerance, and suppressing autoimmune reactions [87,88]. Tregs effectively control the activation, proliferation, and effector functions of key immune cells central to the pathogenesis of multiple sclerosis, such as effector T cells, B cells, and antigen-presenting cells (APCs) [89]. This is achieved through the following mechanisms: secretion of inhibitory cytokines such as the interleukin IL-10; metabolic disruption (Tregs express CD39 and CD73, which suppress Teff cell responses via activation of the adenosine A2A receptor (A2AR)); direct cytolysis mediated by granzyme effects; and inactivation of antigen-presenting cells (Tregs can suppress their maturation via LAG-3/MHC-II interactions and induce IDO, which is mediated by CTLA-4, an immunosuppressive receptor) [89,90]. Furthermore, it has recently been established that Tregs participate in enhancing tissue repair. In the CNS, Tregs have been shown to directly drive remyelination, independent of immunomodulation, through the production of the growth regulator CCN3 [91,92].

A deficiency and/or dysfunction of natural regulatory T cells (Tregs) is a key factor enabling autoreactive lymphocytes to attack myelin in the central nervous system in multiple sclerosis. Consequently, increasing the number and/or restoring the function of Tregs in the patient’s body should suppress the pathological immune response, restore immunological tolerance, and thereby halt or slow disease progression. Myelin-autoreactive T cells are present in both MS patients and healthy individuals, suggesting that their presence alone is insufficient to induce disease and requires immune dysregulation. This may arise from dysfunction of systemic or local immunoregulatory systems, either through genetic predisposition or temporarily as a product of a concomitant infection [93].

Tregs demonstrate unique properties that underscore their potential as viable candidates for cell therapy in multiple sclerosis. Tregs can effectively migrate to target tissues, following which chemoattractant molecules are released at the site of inflammation. The compartmentalization and migration of Tregs appear to be tissue specific, with varying expression of chemokine receptors and integrins facilitating the selective retention and movement of Tregs to sites where regulation is needed [94]. However, the specific migratory pathways and chemokine receptors involved in Treg migration to the CNS are not yet fully understood. Tregs also have a long lifespan in vivo and can self-regulate their number and function depending on the therapeutic need in the target tissue [95]. Tregs do not require direct contact with autoreactive immune cells to exert their suppressive effects, as they are capable of altering the local inflammatory environment through the expression of cell surface receptors and the production of soluble mediators, such as the anti-inflammatory cytokines transforming growth factor TGF-β, IL-10, and IL-35. These cytokines can also promote the emergence of additional subtypes of immunosuppressive cells in a process called “infectious tolerance” [96].

During MS development, Treg dysfunction is characterized by a reduction in Treg number and suppressive activity, an impaired response to myelin autoantigens [58,59], and resistance of effector T cells to regulatory signals. In this context, approaches to generate Tregs in vitro allow the production of functionally competent cells. Their subsequent adoptive transfer into patients offers several advantages: autologous origin (minimizing rejection risk), long-term effects due to their ability for self-regulation in vivo [60], tissue-specific migration (chemotaxis to inflammation sites (CNS)) [61], neuroprotection (stimulation of remyelination) [62], and personalization (possibility of ex vivo modification (e.g., antigen-specific Tregs)) [63].

The administration of in vitro-induced Tregs to patients does not guarantee an absolute effect. The tolerogenic effect of Treg therapy may face the following problems: the presence of Treg dysfunction in patients, phenotype instability, lack of specificity to myelin antigens, risk of systemic immunosuppression, and an unknown optimal dosage of the cell preparation. To address these problems, research is being conducted using ex vivo expansion with IL-2/rapamycin [64], gene editing (e.g., CRISPR to enhance FoxP3), the selection of stable subpopulations (nTreg, Tr1), the use of epigenetic modifiers, the generation of antigen-specific Tregs primed with MBP/MOG/PLP, local administration (intrathecal, intralymphatic), an IL-2 regimen, and dose-dependent studies [97].

Recent studies have suggested impaired function of Tregs obtained from MS patients, as well as unresponsiveness of effector T cells to Treg-mediated suppression. This has implications for autologous Treg transfer, as any deficit in the functional capacity of Tregs derived from MS patients would likely need to be corrected prior to reinfusion for the therapy to be effective [90,98].

Building on promising results from preclinical animal studies, several clinical studies have demonstrated the safety and tolerability of adoptive Treg transfer in conditions such as type 1 diabetes [99], GVHD [100], and amyotrophic lateral sclerosis (ALS) [101], and additional clinical trials are currently underway. Efficacy was demonstrated in ALS, where autologous Tregs expanded in vitro and were administered intravenously with concomitant IL-2, slowing the rate of progression in both the early and late stages of the disease [101].

Interestingly, in patients with amyotrophic lateral sclerosis (ALS), dysregulation of Tregs was also shown, which was reversed after ex vivo expansion with rapamycin/IL-2 and intravenous adoptive transfer of Tregs with concomitant subcutaneous IL-2 injection. However, the mechanisms underlying these phenotypic changes are unknown. An alternative approach would be to use gene editing to remove polymorphic human leukocyte antigens on fully functional Tregs obtained from unrelated patients. This approach is currently being used with adoptive transfer of tumor-infiltrating effector (Teff) cells in some cancers [102,103] (Table 1).

Currently, approaches combining Treg therapy for MS with IL-2 to maintain Tregs in vivo, targeted delivery to the CNS (CCR5-modified Tregs), and the use of Tregs with chimeric antigen receptors (CAR-Tregs) [104] are being tested.

Treg therapy, which combines immunosuppression and neuroprotection, has unique potential for treating MS. However, for clinical implementation, further work is needed on the selection and expansion of functional Tregs, determination of optimal antigen targets (myelin/neuronal peptides), development of strategies to manage Treg plasticity (preventing conversion to Th17), and ensuring targeted delivery to the CNS with minimized systemic effects. Identifying causal epitopes on an individual basis is problematic, complicating the induction of antigen-specific tolerance in MS. Adoptive transfer of polyclonal Tregs with broad antigen specificity may be less technically complex and allow for the administration of a larger number of Tregs, but issues may arise owing to off-target effects on tissue cells. Antigen-specific Tregs may provide more localized suppression of harmful immune responses in the CNS and/or draining lymph nodes in MS. Thus, further research is needed to identify potential driver antigens in MS to achieve the maximum effect of Treg-based therapy.

The risks associated with regulatory T cell (Treg) therapy for multiple sclerosis (MS) are generally considered relatively low compared to conventional immunosuppressants or classical CAR-T therapies. Phase I/II clinical trials confirm a favorable safety profile, with no serious adverse events such as severe infections or cytokine release syndrome reported. However, potential risks do exist, primarily stemming from the biology of Tregs and the specific features of the disease [105].

A key risk is insufficient or short-lived efficacy. Polyclonal Tregs may not adequately migrate across the blood–brain barrier into the central nervous system, particularly following intravenous administration, leading to a limited therapeutic effect and potential disease relapses. Antigen-specific Treg variants are more effective but require precise identification of the relevant autoantigens.

Another significant concern is the plasticity and potential loss of Treg stability. In the inflammatory CNS environment, these cells can convert into proinflammatory phenotypes (e.g., Th17-like), potentially exacerbating the autoimmune process. This is particularly relevant for polyclonal Tregs, where the risk is higher; engineered variants (e.g., CAR-Tregs or Tr1-like cells) demonstrate greater stability.

Systemic immunosuppression poses a lesser risk compared to standard drugs, as Tregs primarily act in a localized and antigen-specific manner. However, polyclonal forms may induce non-specific immune suppression, potentially increasing the long-term risk of infections or malignancies. Clinical data to date have not confirmed a significant rise in infection rates. Persistent challenges with migration and CNS penetration remain substantial; the cells exhibit poor barrier trafficking, and while intrathecal administration may be more effective, it carries additional invasive risks.

For off-the-shelf allogeneic products, such as TRX319, theoretical risks include rejection reactions or graft-versus-host disease. Nonetheless, the inherent immunosuppressive properties of Tr1/Treg cells minimize this risk, and preclinical data indicate safety without the need for prior lymphodepletion.

Finally, long-term effects remain unknown; potential concerns such as oncogenicity, chronic immunosuppression, or impact on neurodegeneration require years of monitoring, which is currently lacking due to the short follow-up periods in existing studies.

In summary, the primary “risk” of Treg therapy to date is not toxicity, but rather the variable clinical efficacy, which drives the development of more targeted and stable cell products.

Therefore, further research is necessary to identify potential driver antigens in multiple sclerosis to maximize the therapeutic efficacy of Treg-based approaches.

### 2.4. CAR-Tregs

CAR-Tregs are genetically modified regulatory T cells expressing chimeric antigen receptors (CARs) specific to myelin antigens (e.g., MOG and MBP). Their key features are antigen specificity (targeting autoreactive T cells in the CNS) [105,106], enhanced suppressive activity (preservation of FoxP3-dependent immune response suppression), and tissue tropism (ability to migrate to inflammation sites) [107].

Natural Tregs suppress excessive immune responses, but in patients with multiple sclerosis (MS), they are often functionally impaired or insufficiently effective. It is hypothesized that genetically engineered Tregs equipped with chimeric antigen receptors (CARs) specific for myelin antigens (e.g., MOG or MBP) could specifically recognize and accumulate at inflammatory sites within the central nervous system (CNS). By overcoming the blood–brain barrier, these cells could suppress the activity of autoreactive T and B cells responsible for myelin damage [106].

Several authors have conducted studies on the application of CAR-Tregs in EAE. Fransson et al. developed anti-MOG CAR-Tregs for EAE, which demonstrated significant immunosuppressive effects in vitro [105]. In vivo, these cells led to the regression of disease manifestations and prevented relapse. Moreover, overall astrocyte proliferation and myelin production were more pronounced in the anti-MOG CAR-Treg treatment group than in the other groups, suggesting a broader impact. Similarly, another preclinical study showed that anti-MOG/MBP CAR-Tregs reduced EAE severity scores and delayed disease progression [97]. Owing to the migratory ability of Tregs, anti-MOG CAR-Tregs can penetrate brain structures, e.g., cerebellar white matter, and reduce EAE activity scores [97]. The application of CAR-Tregs has been well demonstrated in GVHD. MacDonald et al. demonstrated that anti-HLA-A2 CAR-Tregs prevent GVHD development in immunodeficient mice [108]. Furthermore, several preclinical studies have investigated the potential of CAR-Tregs in treating autoimmune diseases such as multiple sclerosis, vitiligo, and inflammatory bowel diseases, all of which have shown improvements in symptoms and serological markers [108].

In perspective, CAR-Tregs could offer additional opportunities to combat autoimmune diseases without causing B-cell depletion. For example, in preclinical models of systemic lupus erythematosus (SLE), anti-CD19 CAR-Tregs were able to suppress B-cell proliferation and activity and limit the generation of autoantibodies, thereby creating a tolerance-inducing microenvironment [109]. However, the efficacy of such approaches in patients with autoimmune diseases remains unknown. Given the plasticity of Tregs, in situ loss of the ability to stimulate tolerance could affect the capacity of CAR-Tregs to induce long-term drug-free remission. In vivo (experimental MS model) and in vitro studies have shown that anti-MOG CAR-Tregs suppress inflammation and demyelination, anti-MBP CAR-Tregs reduce disease severity, and CAR-Tregs inhibit the proliferation of autoreactive T cells [110]. To reduce Treg plasticity (risk of conversion to Th17/Teff cells under the influence of IL-6 and TNF-α), genomic editing (stabilization of FoxP3) and removal of receptors for proinflammatory cytokines are being conducted [111]. The risk of systemic immunosuppression is reduced by the local administration of therapeutic cells (intrathecal, intranasal) and the use of a “suicide gene” (iCasp9) (Table 1).

CAR-Treg therapy is a promising direction for MS treatment, combining antigen-specific suppression of the autoimmune response, neuroprotection (stimulation of remyelination), and personalization (selection of CARs on the basis of the patient’s individual antigen profile). However, their application faces numerous obstacles at various stages of development, stemming from the fundamental mechanisms of CAR-Treg function and the pathogenesis of multiple sclerosis, where cell migration, stability, and specificity play key roles. To date, there is no convincing clinical evidence for the efficacy of CAR-Tregs in MS therapy. Most studies are preclinical. A major challenge is the complexity of identifying suitable target antigens. MS is characterized by multiple autoantigens (e.g., myelin oligodendrocyte glycoprotein—MOG, myelin basic protein—MBP). CAR-Tregs require precise targeting, but CARs recognize only surface antigens, unlike T-cell receptors (TCRs) which can respond to intracellular peptides. Selecting the wrong antigen can lead to therapy failure or off-target effects (impact on healthy tissues). In preclinical models, the antigen specificity does not always cover all autoreactive clones [89,112].

The pronounced plasticity and instability of Tregs also presents a significant barrier. Under the influence of inflammatory cytokines (e.g., IL-6, TNF-α, IFN-γ), Tregs can lose expression of FoxP3—a key transcriptional factor—converting into proinflammatory effector cells (Th17-like). This is particularly relevant in the inflammatory CNS environment of MS, where Tregs from patients already exhibit reduced suppressive activity and increased susceptibility to apoptosis. Strategies such as FoxP3 overexpression or CAR modifications (e.g., incorporating 4-1BB domains) help but do not fully resolve this issue.

Another notable challenge is impaired Treg migration across the blood–brain barrier (BBB). Tregs from MS patients demonstrate reduced migratory capacity under non-inflammatory conditions due to defects in chemokine receptors (e.g., CXCR3, CCR6, CCR8) and adhesion molecules (LFA-1, CD49d). Inflammation (e.g., during relapse) can restore migration, but Tregs do not always reach lesion sites. Intrathecal administration shows better results, whereas systemic delivery is often ineffective. This limits local suppression of inflammation and tissue repair. CAR-Tregs poorly infiltrate tissues, including the CNS, and their survival is IL-2-dependent. In autoimmune diseases, persistence is shorter than in oncology due to the absence of or milder lymphodepletion regimens, leading to a transient therapeutic effect [89].

This problem may potentially be addressed through the development of polyantigenic constructs and gene circuits encoding FoxP3 to stabilize the phenotype of antigen-specific Tregs.

Therapeutic efficacy can also vary depending on the disease stage. CAR-Tregs are effective in early stages by suppressing the initiation of the immune response but are less potent in progressive MS forms, where chronic degeneration and resident memory T cells dominate [113].

Furthermore, Tregs constitute only 5–10% of peripheral CD4+ T cells, and their expansion requires specialized conditions to prevent loss of suppressive function. Surface markers (CD4+ CD25+ CD127low) are not ideal, and FoxP3 is intracellular, complicating cell sorting. Autologous cells from MS patients are often dysfunctional [112].

Risks associated with genetic engineering also exist. Lentiviral vectors carry a risk of insertional mutagenesis and oncogenesis, while non-viral methods (e.g., electroporation) suffer from low efficiency. For “off-the-shelf” (allogeneic) products, TCR/HLA editing is necessary to avoid graft-versus-host disease or host-versus-graft reactions.

### 2.5. CAR-T Cells

CAR-T (chimeric antigen receptor T) cells are genetically modified T lymphocytes programmed to recognize and destroy target cells. In oncology, they are successfully used against B-cell malignancies. Genetically engineered T cells targeting CD19 or BCMA deplete B cells and autoreactive lymphocytes, crossing the blood–brain barrier to locally suppress inflammation within the central nervous system. In autoimmune diseases, including multiple sclerosis (MS), their potential is attributed to (1) targeted depletion of B cells, including autoreactive clones, potentially leading to an “immune reset” [114]; (2) elimination of pathogenic plasma cells producing autoantibodies (e.g., in anti-MOG- or anti-AQP4-mediated MS); and (3) penetration into the CNS, where CAR-T cells can target resident B cells and plasma cells inaccessible to monoclonal antibodies [115] (Table 1).

Currently, key targets for CAR-T-cell therapy in MS include the following antigens: CD19 (B cells), BCMA (plasma cells, plasmablasts), CD38 (plasma cells, early B cells), and MOG/MBP (for CAAR-T cells). Current approaches to CAR-T-cell therapy utilize lentiviral or retroviral vectors for CAR delivery into effector cells. Research is also underway on alternative transduction methods, including the use of lipid nanoparticles (LNPs) loaded with either mRNA encoding the CAR or a CRISPR-Cas9-based gene editing payload [116,117,118]. Furthermore, in vivo CAR approaches are being developed on the basis of the injection of “targeted” LNPs that express receptors preferential for specific cells (such as T cells), where the LNPs can deliver an mRNA payload encoding the CAR. In addition to these production strategies, novel CAR designs, such as chimeric autoantibody receptor (CAAR) T cells [119] and chimeric autoantigen T-cell receptor (CATCR) T cells [120], are being developed for the selective depletion of antigen-specific B cells. These cells express an autoantigen linked to a truncated CAR or TCR, respectively. Autoimmune diseases have a relatively low target antigen load, with primary pathology driven by autoantibodies produced by plasma cells. Therefore, the use of CAR-T-cell therapy to deplete all B cells is excessive and increases the risk of adverse reactions. This drawback can be overcome via the use of CAAR-T cells, whose antigen-binding domains consist of autoantigens. These CAAR-T cells can recognize BCRs and kill autoreactive B cells without affecting normal B cells [121].

Currently, the most widely used CAR-T cells are those that target B cells or plasma cells, as this is the only approach applied in autoimmune diseases thus far. Most CAR-T-cell therapy methods for patients with autoimmune diseases utilize anti-CD19 CAR-T cells. Targeting CD19 offers several advantages, as this cell surface molecule is highly specific for the B-cell lineage and is expressed throughout B-cell differentiation, except on long-lived plasma cells [122]. Thus, targeting CD19+ cells allows for the depletion of mature B cells, including naive and memory B cells and plasmablasts, as well as B-cell precursors, including pro-B and pre-B cells in the bone marrow. CD19 is expressed predominantly on B cells. However, a small fraction of cells in the CNS, known as mural cells, also express CD19 [123]. These cells surround endothelial vessels in the brain and are involved in maintaining the blood–brain barrier. Neurotoxicity can occasionally occur with anti-CD19 CAR-T-cell therapy, and one hypothesis links this adverse effect to the depletion of mural cells combined with increased blood–brain barrier permeability. A second, more general hypothesis suggests that in the context of CAR-T-cell activation, certain cytokines cross the blood–brain barrier and activate microglia [124].

Another promising target for CAR-T cells is CD38, a cell surface protein expressed on plasma cells, early B cells, and subsets of T cells and NK cells [125]. Compared with CD19, targeting CD38 offers a distinct spectrum of target cells. This approach potentially combines the advantages of targeting antibody-producing cells (plasmablasts and plasma cells) and depleting B cells at early differentiation stages without affecting mature B cells, including memory B cells. Given that monoclonal antibodies targeting CD38 appear to have a beneficial effect on SLE [126], CD38 may be an attractive target for cell therapy in autoimmune diseases. Another strategy considered for malignancies involves simultaneous targeting of two different B-cell antigens (such as CD19 and CD22) to prevent antigen “escape,” as malignant B cells are often quite plastic [127]. Whether such strategies are applicable to autoimmune diseases is unknown. Notably, the mutation rate and thus plasticity of malignant B cells are greater than those of autoreactive B cells. Therefore, immune escape associated with loss of the target antigen is more likely in patients with malignancies than in those with autoimmune diseases. Indeed, B cells that relapse after anti-CD19 CAR-T-cell therapy in patients with autoimmune diseases uniformly express CD19 and thus show no signs of immune escape [128].

The use of CAR-T-cell therapy for treating neurological immune-mediated conditions, particularly multiple sclerosis, is not new [129]. Compared with monoclonal antibodies, CAR-T-cell therapy has the advantages of broader depletion of autoreactive B cells, especially those persisting in inflamed tissues such as the central nervous system, and access to lymphoid organs such as deep lymph nodes and the spleen [130]. This approach could lead to the removal of “hard-to-reach” pathogenic B cells and plasmablasts. This is highly relevant for MS patients, given what is known about the disease mechanism and the role of Epstein–Barr virus (EBV) infection and persistent autoreactive B cells—a target for which EBV-associated refractory lymphomas are successfully treated with CAR-T cells [131,132].

In addition to the immunoregulatory effects of CAR-Tregs, anti-CD19 CAR-T cells are also aimed at suppressing EAE development by targeting B cells that contribute to autoantibody production. Gupta et al. [131] reported that anti-CD19 CAR-T cells significantly reduced EAE activity scores, delayed disease onset, and maintained the absence of CD19+ B cells for more than 25 weeks, surpassing the efficacy of CD20 antibodies. However, another experiment yielded opposite results, where injection of anti-CD19 CAR-T cells led to worsening of EAE after 2 weeks, with a broader spectrum of pathological demyelination and axonal loss, despite assisting in the clearance of meningeal B-cell deposits involved in MS immunopathology [132]. This may be associated with the activation of proinflammatory responses that exacerbate chronic systemic inflammation, as well as specific stimulation of the cellular component of the autoimmune response implicated in MS. Therefore, it is important to dynamically assess both the humoral and cellular arms of immunity during the course of MS and its treatment. For CNS autoimmune diseases such as EAE, CAR-based therapy outperforms mAbs because of its ability to migrate into the CNS to exert a therapeutic effect. For example, B cells are completely depleted in the periphery and within the CNS by anti-CD19 CAR-T cells, indirectly confirming their ability to penetrate the CNS [133].

Preclinical studies in an experimental autoimmune encephalomyelitis (EAE) model have shown that anti-MOG CAR-Tregs suppress inflammation and stimulate remyelination [84,85]. Anti-CD19 CAR-T cells have demonstrated conflicting results, ranging from improvement to exacerbation of EAE [86,87].

Alternative targets to CD19 are being considered for the treatment of B-cell-mediated autoimmune disease. BCMA is expressed predominantly on mature B-cell populations, including memory B cells, plasmablasts, and plasma cells. Depleting plasma cells with anti-BCMA CAR-T cells may offer some advantages over anti-CD19 CAR-T-cell therapy. Indeed, anti-BCMA CAR-T cells are currently used to treat multiple myeloma [134]. Isolated depletion of BCMA-expressing cells has been shown to be effective in treating autoimmune visual impairment associated with neuromyelitis and anti-SRP necrotizing myopathy [135].

Although targeting plasma cells via BCMA can eliminate autoantibody-producing plasma cells, this approach also significantly impacts plasma cells that produce protective humoral responses against viruses and bacteria developed after vaccination and infections. Overall, dual targeting of CD19 and BCMA or single targeting of BCMA may be applicable in subgroups of patients with autoimmune diseases where autoantibody production is predominantly driven by plasma cells. Such patients could be identified by assessing those who relapse after anti-CD19 CAR-T-cell therapy, potentially due to persistent autoantibody production.

Limited clinical trials (Phase I) have shown that the use of anti-BCMA CAR-T cells for therapy of recurrent antibody-associated inflammatory diseases of the nervous system (NCT04561557) resulted in remission in 11 of 12 patients at 5.5 months [135]; the use of anti-CD19 CAR-T cells in progressive MS (NCT06138132) led to a reduction in intrathecal IgG in one patient and stabilization of the patient’s condition on the EDSS in another; for anti-CD19 CAR-T cells (KYV-101) (NCT06451159), a safety assessment was conducted in refractory MS.

A number of clinical trials are currently registered:

‑ A phase I, multicenter, noncomparative study evaluating the safety, tolerability, efficacy, and effectiveness of the drug CC-97540 (anti-CD19 CAR-T cells) in participants with relapsing or progressive forms of multiple sclerosis (NCT06220201);
‑ A study of anti-CD19 CAR-T-cell therapy in patients with nonrelapsing and progressive forms of multiple sclerosis: an open-label phase I study conducted at a single center (NCT06138132);
‑ A phase I, open-label, single-center study of KYV-101, a CD19 CAR-T-cell therapy, in participants with treatment-refractory progressive multiple sclerosis (NCT06451159);
‑ A phase II, open-label, randomized, multicenter study of KYV-101, an autologous fully human anti-CD19 CAR-T-cell therapy in patients with refractory primary and secondary progressive multiple sclerosis (KYSA-7) (NCT06384976);
‑ A KYSA-7 (Phase 2) study of anti-CD19 CAR-T cells in 12 patients with progressive multiple sclerosis (NCT06138132).

The application of CAR-T cells is associated with challenges due to neurotoxicity, T-cell plasticity, heterogeneity of MS forms, and target antigens. The most frequent adverse reactions associated with CAR-T-cell therapy, particularly targeting CD19, are cytokine release syndrome (“cytokine storm”), immune effector cell-associated neurotoxicity syndrome (ICANS), and hematotoxicity. When anti-BCMA CAR-T cells are used, other characteristic neurological complications have been described, including Parkinsonism, cranial nerve palsies, and peripheral neuropathy, which are not uncommon and can be irreversible [136]. The safety of using anti-BCMA CAR-T cells for treating immune-mediated neurological conditions is currently being carefully studied [136]. To address these problems, optimization of the cell preparation dose, premedication with tocilizumab [137], editing of the FoxP3 gene, removal of proinflammatory receptors, and personalization of target antigens (e.g., EBV-specific CAR-T cells [138,139]) are being conducted.

Several potential directions for the development of CAR-T-cell therapy for MS should also be noted:

‑ CAAR-T (chimeric autoantibody receptor T cells)—T cells expressing autoantigens (e.g., MOG) for selective destruction of autoreactive B cells, reducing the risk of systemic immunosuppression.
‑ CAR-NK cells have a shorter lifespan and a lower risk of “cytokine storm.”
‑ EBV-specific CAR-T cells target B cells infected with the Epstein–Barr virus (a possible trigger for MS development).

Currently, developments are underway to improve CAR-T-cell production technology. A more specific approach involves the use of so-called chimeric autoantibody receptor (CAAR) T cells [137]. Unlike CARs, which identify and attach to their target via an extracellular antibody fragment, CAARs direct the cytotoxic effects of T cells only to autoantibody-producing B cells, offering the advantage of reducing the risk of general immunosuppression. Since CAAR-T cells also bind circulating autoantibodies, CAARs can become saturated, so a greater number of infused CAAR-T cells than CAR-T cells may be required for efficacy. An additional potential risk of CAAR T-cell therapy is the so-called “tip of the iceberg” phenomenon in autoimmunity, which implies that not all autoantibodies contributing to pathology are always identified. To overcome this problem, at least partially, CAAR-T cells with multiple specificities would be useful. However, different neuroimmunological disorders may benefit differently from various approaches. Therefore, we must await data from current and future clinical trials [137].

As part of the development of approaches to induce antigen-specific tolerance in autoimmune diseases and MS, a search for new antigenic targets is underway. The risk of developing MS is associated with the presence of a latent infection—the Epstein–Barr virus (EBV). It is unique among viruses infecting humans in its ability to infect, activate B lymphocytes, and then persist as a latent infection in these cells. It infects approximately 90% of the adult population worldwide and persists for the lifetime of the infected individual in terms of B cells, plasma cells, and tonsillar epithelial cells. The number of EBV-infected cells is normally under strict immune control, particularly by EBV-specific cytotoxic CD8+ T cells that kill proliferating and lytically infected cells [140]. In 2003, Pender M.P. et al. hypothesized that a defect in CD8+ T-cell control over EBV predisposes patients to MS, allowing EBV-infected autoreactive B cells and plasma cells to accumulate in the CNS [140]. This hypothesis was supported by data on the accumulation of EBV-infected B cells and plasma cells in the brain in MS; however, other studies failed to find evidence of EBV infection in the brain [141]. An implication of this hypothesis is that EBV-specific T-cell therapy should kill EBV-infected B cells in the CNS and thereby prevent disease progression and lead to clinical improvement [131,142]. EBV-specific CD8+ T-cell lines were obtained from MS patients via in vitro stimulation with EBV antigens [132], followed by studies of EBV-specific T-cell therapy [143,144]. EBV-infected B cells in the brain in multiple sclerosis express the same 3 Epstein–Barr virus proteins [144]. Initial clinical studies of this approach have been conducted; therapy of an MS patient with Epstein–Barr virus-specific T-cell therapy was accompanied by clinical improvement and a reduction in CSF IgG production, which persisted for 3.5 years after T-cell therapy [131,142]. Therapy with autologous Epstein–Barr virus-specific T cells was well tolerated by patients, with no serious side effects.

Thus, CAR-T-cell therapy in MS is a promising but complex approach that requires optimization of safety (minimizing neurotoxicity and infectious risk), personalization (selecting targets (CD19/BCMA/CD38) on the basis of the patient’s immune profile), and combination with other approaches (with IL-2 to maintain Tregs, with anti-CD20 for synergistic B-cell depletion).

### 2.6. TCR-T Cells

TCR-T-cell therapy represents a promising direction in the treatment of multiple sclerosis and is based on the use of genetically modified T lymphocytes with specific T-cell receptors. Unlike CAR-T-cell therapy, which uses artificial chimeric receptors, TCR-T-cell therapy is based on natural mechanisms of antigen recognition in the context of major histocompatibility complex (MHC) molecules [145] (Table 1).

The development of MS is based on autoreactive T cells that recognize myelin antigens (MBP, MOG, and PLP), penetrate the blood–brain barrier, and initiate a cascade of inflammatory reactions. Accordingly, TCR-T-cell therapy for MS offers two principal approaches: suppression of pathogenic autoreactive T cells and activation of regulatory T-cell populations. Currently, preclinical studies are being conducted on the use of TCR T cells for the therapy of a number of autoimmune processes (type I diabetes) [146], but for multiple sclerosis, such work is limited and based on vaccination with TCR peptides [147].

Modern strategies for the treatment of multiple sclerosis using T cells with a T cell receptor have several main directions:

- TCR peptide vaccination (TCR vaccinotherapy): This approach utilizes synthetic peptides corresponding to hypervariable regions of the TCR expressed on pathogenic autoreactive T cells (e.g., Vβ5.2 or Vβ6 chains). Administration of these peptides elicits an immune response against the pathogenic clones, thereby reducing their frequency [148]. Clinical studies have demonstrated decreased relapse rates and disease stabilization in some patients, with “responders” showing diminished reactivity to myelin antigens and no serious adverse effects. However, efficacy has been variable across patients [149,150].
- TCR-engineered regulatory T cells (TCR-Tregs): Tregs are modified to express TCRs specific for MS-relevant autoantigens (e.g., MBP), thereby enhancing antigen-specific suppression [112].
- TCR-like chimeric receptors and hybrid constructs: These include TCR-mimetic CARs (sometimes termed CATCRs) and combined CAR-T/Treg platforms designed to recognize a broader range of antigenic epitopes, including intracellular antigens presented in complex with MHC molecules [137,151,152].
- Recombinant TCR ligands (RTLs): RTLs are engineered partial TCR molecules that mimic the natural TCR structure but are optimized for binding specific peptide–MHC complexes. Due to their biochemical stability and structural homology to human MHC class II, RTLs offer potential as epitope-specific therapeutics for CD4+ T cell-mediated autoimmunity [153,154].

Additional advancements include the following:

- Therapy personalization: TCR repertoire sequencing enables identification of patient-specific autoantigen profiles, facilitating the development of highly individualized T cell products [112,155].
- Safety and combination approaches: Efforts focus on mitigating risks such as cytokine release syndrome (CRS), immune effector cell-associated neurotoxicity syndrome (ICANS), secondary infections, and oncogenesis, alongside evaluation of efficacy in preclinical and clinical settings [92].

Despite these advances, TCR-T cell therapy faces several challenges: safety concerns (e.g., oncogenesis risk from viral vectors, CRS, and off-target cross-reactivity); efficacy limitations (e.g., antigen heterogeneity, immune escape mechanisms, and immunosuppressive CNS microenvironment); and manufacturing hurdles (e.g., complexity of personalized production, high costs under good manufacturing practice (GMP) conditions, and standardization) [156].

In summary, TCR-T cell therapy offers a novel paradigm for MS treatment by providing high antigen specificity, the potential to generate antigen-specific regulatory cells, and durable therapeutic effects. Compared with CAR-T cells, TCR-T approaches benefit from physiological antigen recognition, reduced risk of severe cytokine storms, and the capacity to target intracellular antigens.

Future directions include optimization of TCR affinity, enhancement of T cell persistence, and development of off-the-shelf allogeneic products. Notwithstanding current technical challenges, TCR-T cell therapy holds promise as a key component of personalized treatment strategies for MS, particularly in progressive forms, within the coming decade.

## 3. Conclusions

The present analysis of immunotherapeutic strategies for multiple sclerosis (MS) illustrates a dynamic and rapidly evolving research field at various stages of clinical maturity.

The most promising and rapidly advancing direction is CAR-T cell therapy, which in early-phase clinical trials for progressive MS has demonstrated modulation of key pathogenic mechanisms, including depletion of CNS B-cells and reduction in neuroinflammation. Despite its potential to induce long-term remission, further optimization of dosing, management of toxicity, and confirmation of neuroprotective effects are required. The translation of this approach into clinical practice depends on its extension to relapsing forms and the development of combination regimens with regulatory cell populations, such as Tregs. In turn, while TCR-T cell therapy offers a pathway towards antigen-specific immune tolerance, it remains largely in the preclinical stage of development, facing fundamental challenges related to the identification of stable myelin epitopes and the minimization of cross-reactivity.

Among more established methods, autologous hematopoietic stem cell transplantation has confirmed high efficacy in aggressive forms of MS. However, its widespread application is limited by toxicity, driving the development of less intensive, non-myeloablative conditioning protocols. Mesenchymal stromal cell therapy is characterized by a favorable safety profile and promising neuroprotective properties, yet exhibits variable clinical efficacy. This underscores an urgent need for standardization of manufacturing processes and clear biomarkers of response. Tolerance-inducing approaches (e.g., tolerogenic dendritic cells, Tr1/Treg-like cells) are in early stages of clinical validation, demonstrating anticipated immunomodulatory effects but requiring solutions to challenges related to phenotype stability and optimal in vivo delivery.

Thus, the future of MS immunotherapy lies in the realm of personalized medicine and rational combination strategies, particularly for treatment-resistant progressive forms. For all modalities reviewed, progression to a higher level of evidence will depend on conducting large-scale randomized controlled trials with extended follow-up. This is essential for a comprehensive assessment of long-term efficacy, safety, and for defining optimal positioning within MS treatment algorithms.

## 4. Future Perspectives

The prospects for personalized cell therapy in multiple sclerosis (MS) appear promising, particularly given the disease’s heterogeneity, where standard immunomodulators are often insufficient for progressive forms. Personalization involves tailoring treatment to an individual patient’s characteristics: autoantigen profile (e.g., MBP, MOG, PLP), genetic factors (HLA alleles), inflammatory biomarkers, and immune status. This approach aims to achieve specific immune tolerance while minimizing systemic immunosuppression and associated risks.

One of the most dynamic fields is that of chimeric antigen receptor T cells (CAR-T). In 2025, anti-CD19 CAR-T therapies for profound B-cell depletion—key drivers of chronic inflammation (e.g., KYV-101, CC-97540, obecabtagene autoleucel)—are advancing actively. Phase 1 clinical trials demonstrate safety, preliminary efficacy, and signs of remission in patients with progressive MS, including initial applications of allogeneic variants.

Future personalization may involve a shift towards antigen-specific CAR-Tregs (regulatory T cells engineered with CARs targeting myelin antigens). This would enable selective suppression of autoreactive cells and induce long-term tolerance without broad immune suppression. Companies such as PolTreg are advancing such approaches in Phase 1 trials, with the potential to “reset” the immune system.

Autologous tolerogenic dendritic cells (tolDCs), loaded with individual myelin peptides and conditioned with immunomodulators (e.g., vitamin D3 or IL-10), induce anergy in autoreactive T cells and promote Treg expansion. Phase I/II clinical trials confirm their safety and the stability of the effect. Future prospects include personalizing the antigenic repertoire to match a patient’s epitope spreading, combining this approach with other therapies, and implementing repeated administrations to maintain tolerance.

For stem cell-based approaches (HSCs, MSCs), further clinical use is justified and will be linked to refining protocols for their isolation and administration in MS patients.

Other innovations include expanded allogeneic Tregs (e.g., TRX319) or Tr1 cells for tolerance induction; integration with biomarkers (neurofilament light chain, MRI activity) for patient stratification and monitoring; and combined strategies incorporating genetic engineering and artificial intelligence for predictive personalization.

Antigen-specific therapy utilizing T-cell receptors (TCRs) for MS represents a promising alternative to conventional treatments and is among the most relevant current immunological research topics. Progress in this area includes developing strategies to deplete cytotoxic T cells and applying TCR-modified regulatory T cells (TCR-Tregs). Despite advances, further development requires addressing key challenges and leveraging new opportunities. While much foundational data on TCR-based therapy has been derived from animal models replicating key aspects of MS pathogenesis, these models are limited in fully capturing human disease heterogeneity and progression. Therefore, clinical trials in patients are essential to comprehensively assess the efficacy and safety of this cell therapy.

To optimize tolerance induction strategies, it is crucial to enhance the phenotypic stability of TCR-Tregs and further investigate the therapeutic potential of CD8+ Tregs, particularly those modified with TCRs, given their ability to infiltrate inflamed tissues and provide local suppression of pathogenic immune responses. A synergistic approach combining multiple antigen-specific strategies—such as TCR-Tregs targeting different autoantigens, or integrating TCR-Treg therapy with other immunomodulatory agents—may ultimately prove most effective. Furthermore, ongoing research is needed not only to further elucidate the complex mechanisms of action of TCR-Tregs but also to comprehensively characterize their impact on other immune cell populations and evaluate their long-term therapeutic potential.

In our view, TCR-based antigen-specific therapy holds transformative potential for MS treatment, shifting the paradigm from pharmacological management to the induction of cellular tolerance. Despite persistent challenges, such as optimizing TCR affinity and Treg stability, the integration of human-derived autoantigen data with advanced tools like pMHC multimers could accelerate clinical translation. We anticipate the initiation of the first clinical trials of TCR-Tregs within the next 5–10 years, potentially enabling long-term remission without systemic risks. However, to overcome existing limitations, rigorous safety monitoring and the development of multi-antigen targeted therapies will be essential.

## 5. Literature Search Strategy

A systematic search of the peer-reviewed scientific literature was conducted for the present review using the following electronic databases: PubMed/MEDLINE, Scopus, and Web of Science. These databases were selected as the most relevant sources for medical literature in neurology and regenerative medicine, providing broad coverage of clinical studies, meta-analyses, and reviews.

The search encompassed publications from January 2000 to December 2025. The choice of the start date is justified by the significant increase in research on cell therapy for multiple sclerosis (MS) in the early 2000s, coinciding with the advent of the first clinical trials involving stem cells.

The search strategy was developed using a combination of Medical Subject Headings (MeSH) terms and free-text keywords to enhance sensitivity and specificity. The main keywords included the following: “multiple sclerosis” OR “disseminated sclerosis” OR “MS” OR “pacceянный cклepoз” (for Russian-language sources) AND “cellular therapy” OR “stem cell therapy” OR “stem cell transplantation” OR “mesenchymal stem cells” OR “hematopoietic stem cells” OR “MSC” OR “HSC” OR “autologous hematopoietic stem cell transplantation” OR “AHSCT”. Additional combinations used were “pacceянный cклepoз” OR “клeтoчнaя тepaпия” OR “cтвoлoвыe клeтки” OR “дeндpитныe клeтки” OR “T peгyлятopныe клeтки” OR “CAR” OR “TCR” OR “peцeптop T-клeтoк” OR “aнтигeн-cпeциφичecкий” OR “CAR-Treg” OR “TCR-Treg” OR “индyкция тoлepaнтнocти” OR “ayтopeaктивнaя T-клeткa” OR “миeлин”. Supplemental terms included “regenerative medicine” OR “cell-based therapy” OR “T peгyлятopныe клeтки” OR “CAR” OR “TCR” AND “efficacy” OR “safety” OR “clinical trial”.

Inclusion criteria were as follows: (1) publications in the English language; (2) original research or reviews on cell-based immunotherapy involving stem cells or antigen-specific immunotherapy in experimental autoimmune encephalomyelitis models or in human patients with multiple sclerosis; (3) a focus on T-cell modulation, Treg engineering, or autoantigen identification. Exclusion criteria comprised animal models of autoimmunity unrelated to MS; therapeutic methods not utilizing cellular technologies; and studies lacking information on disease mechanisms. A total of 158 references were selected based on relevance, impact, and novelty, with priority given to human-derived data where available. The search was supplemented by cross-referencing key reviews and manual screening of seminal works.

## 6. Limitations of the Review

While this review aims to present current information on cellular technologies and therapeutic approaches for multiple sclerosis, several limitations should be acknowledged. First, the majority of contemporary mechanistic data on genetically engineered T cells and regulatory T cell therapy is derived from preclinical studies, which may not fully capture the heterogeneity of the human disease. Second, the rapid advancement of gene engineering technologies for CAR- and TCR-T cells, genome editing, and the discovery of neoantigens means that new findings may not be fully captured by the publication date. Finally, given that the antigenic landscape of multiple sclerosis and the clinical translation of antigen-specific cell therapies are still evolving, some interpretations presented here should be considered preliminary. Further research, including future systematic reviews and meta-analyses, will help clarify these aspects and strengthen the evidence base required to refine and validate the discussed therapeutic concepts.

## Figures and Tables

**Table 1:** Cellular immunotherapy approaches for multiple sclerosis: features, advantages, and disadvantages.

Cellular Immunotherapy Approaches	Features and Disadvantages	Advantages
**Hematopoietic and Mesenchymal Stem Cells**	‑ requires myeloablative conditioning with high-dose chemotherapy to destroy disease-associated cells; ‑ requires treatment for graft-versus-host disease, ‑ risk of systemic immunosuppression.	‑ low immunogenicity; ‑ versatility (multiple sources: bone marrow, adipose tissue, umbilical cord), relative ease of isolation; ‑ tropism toward sites of inflammation; ‑ regenerative potential
**Tolerogenic Dendritic Cells**	‑ requirements for in vitro culture conditions, ‑ induction of tolerogenic properties of dendritic cells, ‑ risk of loss of tolerogenic properties of dendritic cells and acquisition of an immunogenic phenotype with the production of proinflammatory cytokines and an encephalitogenic effect. ‑ selection of an autoantigen to suppress the antigen-specific reaction.	‑ activates natural mechanisms of immune regulation, ‑ suppression of the autoimmune cellular response against a specific autoantigen (antigen specificity), ‑ ability to use various constructs (combinations) based on DNA, RNA, and/or peptides as antigen material for DC priming. ‑ low risk of developing severe adverse systemic reactions.
**Tregs**	‑ instability of the Treg phenotype, ‑ the presence of Treg dysfunction in patients with multiple sclerosis, ‑ lack of specificity for myelin antigens, ‑ risk of systemic immunosuppression.	‑ can exert a suppressive effect on autoreactive cells without directly interacting with them through receptor expression and cytokine production; ‑ tissue-specific migration (CNS); ‑ neuroprotection (stimulation of remyelination); ‑ antigen specificity; ‑ low risk of developing severe adverse systemic reactions; ‑ the possibility of using approaches using genetic technologies.
**CAR-Tregs**	‑ a relatively complex technology, ‑ risk of conversion to Th17/Teff cells under the influence of proinflammatory cytokines in the patient’s body, ‑ risk of systemic immunosuppression.	‑ genetically modified Tregs, ‑ antigen specificity, ‑ enhanced suppressor activity, ‑ stimulation of remyelination, ‑ tissue tropism (migration to the site of inflammation), ‑ high therapeutic efficacy
**CAR-T cells**	‑ a combination of antigen specificity and T-cell cytotoxicity ‑ low-dose lymphodepletion chemotherapy directed at differentiation is required ‑ CRS (cytokine release syndrome) ‑ ICANS (immune effector cell-associated neurotoxicity syndrome) ‑ primary targets: CD19, BCMA (B-cell maturation antigen), CD38, myelin-related antigens.	‑ MHC-independent antigen recognition. ‑ high therapeutic efficacy
**TCR-T cells**	‑ The targets are myelin-derived antigens (MOG (Myelin Oligodendrocyte glycoprotein), MBP (Myelin Basic Protein)) and viral antigens (EBV (Epstein–Barr Virus))	‑ antigen-specific, ‑ low risk of non-specific immune suppression due to the interaction of the MHC peptide with the TCR. ‑ lower risk of cytokine release syndrome (CRS) and ICANS. ‑ high therapeutic efficacy

## Data Availability

No new data were created or analyzed in this study. Data sharing is not applicable to this article.

## Abbreviations

The following abbreviations are used in this manuscript:

AFACTT	Action to focus and accelerate cell-based tolerance-inducing therapies
AHSCT	Autologous hematopoietic stem cell transplantation
ALS	amyotrophic lateral sclerosis
APCs	Antigen-presenting cells
BDNF	Brain-derived neurotrophic factor
BCMA	B-cell maturation antigen
CAR	Chimeric antigen receptor
CD	Cluster of differentiation
CNS	Central nervous system
CRS	Cytokine release syndrome
DCs	Dendritic cells
DMTs	Disease-modifying therapies
EAE	Experimental autoimmune encephalomyelitis
EDSS	Expanded disability status scale
EBV	Epstein–Barr virus
G-CSF	Granulocyte colony-stimulating factor
GVHD	Graft-versus-host disease
GMP	Good manufacturing practice
ICANS	Immune effector cell-associated neurotoxicity syndrome
HSCs	Hematopoietic stem cells
LAG3	Lymphocyte-activating gene 3
LIF	Leukemia inhibitory factor
LNPs	Lipid nanoparticles
MBP	Myelin basic protein
MS	Multiple sclerosis
MSCs	Mesenchymal stromal cells
MESEMS	Mesenchymal stem cells for multiple sclerosis
MNCs	Mononuclear cells
MITAP	Minimal information about tolerogenic antigen-presenting cells
MHC	Major histocompatibility complex
MOG	Myelin oligodendrocyte glycoprotein
NMOSD	Neuromyelitis optica spectrum disorder
NSCs	Neural stem cells
NGF	Nerve growth factor
PLP	Proteolipid protein
RRMS	Relapsing–remitting multiple sclerosis
SLE	Systemic lupus erythematosus
Tregs	regulatory T cells
tolDCs	Tolerogenic DCs
Teff	Tumor-infiltrating effector
TCR	T cell receptor
